# Long-term impact of intrauterine fetal death on quality of life and depression: a case–control study

**DOI:** 10.1186/1471-2393-12-43

**Published:** 2012-06-07

**Authors:** Ida Kathrine Gravensteen, Linda Bjørk Helgadottir, Eva-Marie Jacobsen, Per Morten Sandset, Øivind Ekeberg

**Affiliations:** 1 Department of Haematology, Oslo University Hospital, Ulleval, Box 4950, Nydalen, N-0424, Oslo, Norway; 2 Department of Obstetrics and Gynaecology, Oslo University Hospital, Ulleval, Oslo, Norway; 3 Department of Acute Medicine, Oslo University Hospital, Ulleval, Oslo, Norway; 4 Department of Behavioural Sciences in Medicine, University of Oslo, Oslo, Norway; 5 Institute of Clinical Medicine, University of Oslo, Oslo, Norway

**Keywords:** Intrauterine fetal death, Quality of life, Depression, Well-being, Socioeconomic status

## Abstract

**Background:**

Intrauterine fetal death (IUFD) is a serious incidence that has been shown to impact mothers’ psychological well-being in the short-term. Long-term quality of life (QOL) and depression after IUFD is not known. This study aimed to determine the association between intrauterine fetal death and long-term QOL, well-being, and depression.

**Methods:**

Analyses were performed on collected data among 106 women with a history of intrauterine fetal death (IUFD) and 262 women with live births, 5–18 years after the event. Univariable and multivariable linear and logistic regression models were used to quantify the association between previous fetal death and long-term QOL, well-being and depression. QOL was assessed using the QOL Index (QLI), symptoms of depression using the Center for Epidemiological Studies Depression Scale (CES-D), and subjective well-being using the General Health Questionnaire 20 (GHQ-20).

**Results:**

More of the cases had characteristics associated with lower socioeconomic status and did not rate their health as good as did the controls. The QLI health and functioning subscale score was slightly but significantly lower in the cases than in the controls (22.3. vs 23.5, *P* = .023). The CES-D depressed affect subscale score (2.0 vs 1.0, *P* = 0.004) and the CES-D global score (7.4 vs 5.0, *P* = .017) were higher in the cases. Subjective well-being did not differ between groups (20.6 vs 19.4, *P* = .094). After adjusting for demographic and health-related variables, IUFD was not associated with global QOL (*P* = .674), subjective well-being (*P* = .700), or global depression score (adjusted odds ratio = 0.77, 95% confidence interval 0.37–1.57).

**Conclusions:**

Women with previous IUFD, of which the majority have received short-term interventions, share the same level of long-term QOL, well-being and global depression as women with live births only, when adjusted for possible confounders.

**Trial registration:**

The study was registered at http://www.clinicaltrials.gov, with registration number NCT 00856076.

## Background

Intrauterine fetal death (IUFD) is a serious complication of pregnancy that influences women’s short-term psychological well-being [1] and increases the risk of experiencing anxiety and depression during the first following months compared with women with a live birth [2]. The risk of experiencing depression and posttraumatic stress disorder (PTSD) is prevalent during the next pregnancy, particularly when conception occurs soon after the loss [3,4]. Few studies have assessed the association between IUFD and the risk of long-term psychological distress. A Swedish 3-year follow-up study reported that women who had experienced stillbirth were twice as likely to experience frequent anxiety symptoms compared with women with a live birth [5]. The Maternal Observations and Memories of Stillbirth study (n = 577) reported that women with a recent loss (<1 year) had more symptoms of depression than did those who had experienced a more distant loss (>1 year), and that the level of anxiety symptoms decreased after two years [6]. In a cohort of women who were initially assessed during a pregnancy subsequent to IUFD, together with controls, Turton et al. found no differences in the levels of PTSD and depression 6–8 years after the birth of the next child [7]. We found no long-term follow-up studies after IUFD that included measures of quality of life (QOL) and general health, and the long-term impact on psychological well-being remains uncertain.

The main objective of this study was to estimate long-term QOL, depression, and well-being in women having experienced an IUFD compared with women with live births only.

## Methods

**Figure 1:**
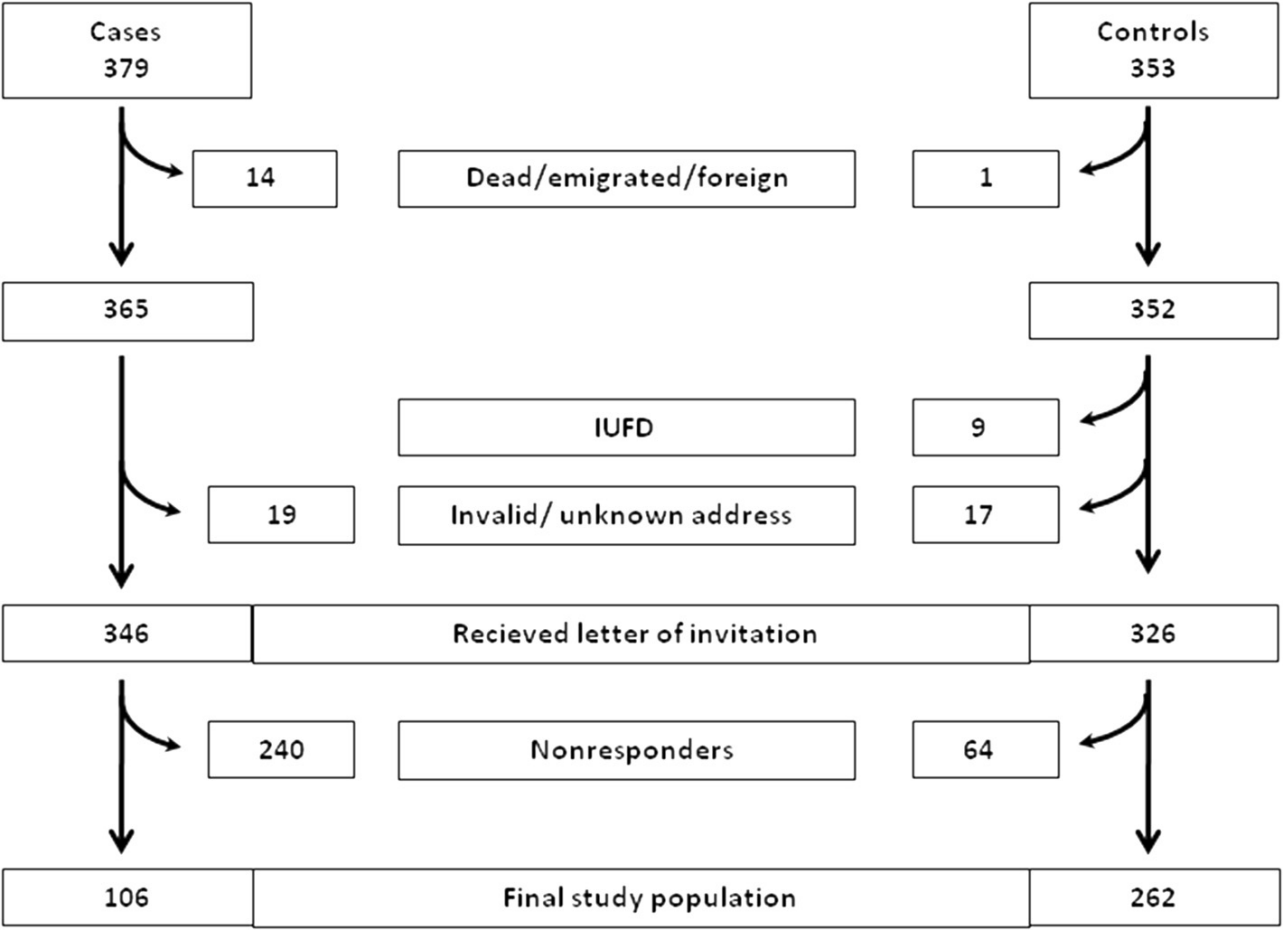
Flowchart for the selection of cases and controls.

The study is a hospital-based case–control study. Women with a diagnosis of IUFD at Oslo University Hospital, Ulleval, Oslo, Norway, and Akershus University Hospital, Lorenskog, Norway, in the time frame January 1, 1990, through December 31, 2003, were identified through the hospitals’ administrative systems. Both Oslo University Hospital, Ulleval and Akershus University Hospital are specialized maternity hospitals. The cases were identified by searching for relevant World Health Organization International Classification of Diseases codes, versions 9 or 10. We identified 439 possible cases of IUFD, defined as fetal death at ≥23 gestational weeks or birth weight >500 g. After reviewing the medical records, we excluded 49 cases wrongly diagnosed, eight with non-retrievable records, and three with triplet pregnancies, leaving 379 women with a verified diagnosis of IUFD in singleton or twin pregnancies. A total of 346 women received a postal invitation to participate in the study. After two reminders, 106 (31%) agreed to participate (Figure 1).

The control group comprised 1,092 women who had live births at Oslo University Hospital, Ulleval, between 1990 and 2003 and who had previously been invited to participate as controls in another arm of the study, the Venous Thromboembolism in Pregnancy (VIP) study [8]. A total of 353 (32%) controls participated in the VIP study in 2006, and at that time they all consented to receiving a new invitation to participate in this arm of the study on a later occasion. A total of 326 of the 353 controls received a postal invitation for the present study in 2008, and 262 (80%) agreed to participate (Figure 1). The final participants constituted 24% of the original control group. All data were collected in 2008–9. The cases and controls who agreed to participate completed a comprehensive questionnaire. The study questionnaire obtained information on demographic, pregnancy, and health-related variables, and three scales measuring QOL, symptoms of depression, and well-being. The questionnaire was optically scanned and the data were transferred electronically to the project database. All the extracted data were manually verified for any scanning errors.

Demographic variables assessed were age, marital status, education, occupational status, household income in Norwegian kroner (NOK), self-reported assessment of personal finances and difficulties in paying bills, body mass index (BMI), smoking, frequency of alcohol consumption, and physical exercise. Pregnancy-related variables assessed were number of pregnancies, number of live-born children, miscarriages and provoked abortions and whether the women felt they had obtained the number of children they wished for.

Health-related variables assessed were pain, sick leave the previous 12 months, physical and mental exhaustion from work and subjective rating of own health. Co-morbidity for the previous 12 months was given as a yes/no response to the following disease categories; cardiovascular disease, hypertension, pulmonary disease, gastrointestinal disease, constipation, kidney/urinary disease, migraine/headache, musculoskeletal disease, cancer, systemic lupus erythematosus (SLE), skin disease, thyroid disease, diabetes, allergy. The disease categories were totalled to give a sum score as there was a trend of more disease in the main categories in the case group. The count in each disease category, however, was too low to reach statistical significance. As the impact of several chronic diseases may be comparable, we chose to sum up the numbers to adjust for this potentially significant factor.

We had information from medical records on demographic and clinical factors of all eligible participants at the time of the index pregnancy. The data included delivery hospital, gestational age, date of index delivery, maternal age, parity, and marital status. These variables were compared between responders and nonresponders in order to assess the risk of selection bias.

For evaluation of QOL, the Ferrans and Powers QOL Index (QLI) was chosen. It is generic and provides a global, multidimensional view of QOL [9]. The questionnaire also includes the individual’s set of values by assessing subjective satisfaction with various items and the importance of these items to the participant [10]. The QLI is used worldwide and has been translated into Norwegian and tested for reliability and validity [11]. The factor structure comprises four subdomains: 1) health/functioning (14 items), 2) psychological/spiritual (seven items), 3) socioeconomic (nine items), and 4) family (four items). The Cronbach’s alpha values were .70–.91 for the four subdomains of QLI in our study and .94 for the global score. We used the original 6-point Likert scale of 0–5 to score the 34 items of the generic version II, giving a total range of 0–30, with high values denoting better QOL. The score was calculated by adjusting the responses on satisfaction with an item by the importance of the same item to the subject. The entire scale was considered missing if more than five items were missing in either the satisfaction responses or the importance responses [10]. Scores for the QLI were computed using standard scoring algorithms available at http://www.uic.edu/orgs/qli. (The QLI has been revised recently and the scoring algorithms presented on the web-side are applicable for the QLI version III, The QLI version II was used in the present study.)

Subjective well-being was assessed using the General Health Questionnaire (GHQ-20) [12]. The 20-item version has been translated into Norwegian and tested for validity and reliability [13,14]. The scale comprises questions about general health focusing on non-psychotic psychological health and has a content that corresponds closely with the concept of subjective well-being.

The scale includes both positively and negatively phrased items. Each item is rated on a 4-point (0–3) Likert scale (less than usual, no more than usual, rather more than usual, or much more than usual), giving a range of 0–60 with high values denoting greater distress. Cronbach’s alpha in our study was .88. The entire scale was considered missing if more than four items were missing.

To assess the level of depressive symptoms, we used the Center for Epidemiological Studies Depression Scale (CES-D Scale) [15]. The scale comprises 20 items on a 4-point scale ranging from 0 (“rarely”) to 3 (“all the time”). The total range is 0–60, and a global score of 16 or more indicates a case level depression [15]. The factor structure comprises four subdomains: 1) depressed affect (seven items), 2) positive affect (four items), 3) somatic affect (seven items), and 4) interpersonal (two items). The Cronbach’s alpha values were .69–.83 for the four subscales in our study and .87 for the global score. The entire CES-D scale was considered missing if more than four items were missing [15].

Pearson’s chi-square or Fisher’s exact test were used to compare categorical variables. The QLI subscales of health and functioning, socioeconomic, and psychological/spiritual, the QLI global score, and the GHQ-20 were assessed using *t* tests. The Mann–Whitney *U* test was used to asses all CES-D subscales, the CES-D global score, and the QLI family subscale because the distributions were markedly skewed.

When making a power analysis we considered a minimum difference of 2 (50% of SD) units on global QLI in order to maintain clinical relevance of our results [16]. When performing an independent samples *t*-test with a 5% type I error and a sample size of 106 cases and 262 controls (SD = 4.0), the design had a >95% power to detect such a difference.

For the QLI global score, GHQ-20, and CES-D global score, multivariable analyses were used to adjust for possible confounders among sociodemographic and health related factors that may influence these outcomes. For the multivariable analysis of the QLI and GHQ global scores, we applied a linear regression model. For the multivariable analysis of the CES-D, the global score was dichotomised at the predefined cut-off value of 16, and a logistic regression model was applied. Sociodemographic and health related variables that showed uneven distribution between the cases and controls (*P* < .2), were associated with the outcome variable (*P* < .2), and not intercorrelated (correlation coefficient < .7), were included in the multivariable analyses. Variables with <10 subjects in at least one of the categories were not included in the models. Forward variable selection was used to identify predictors of QLI, GHQ, and CES-D scores above the cut-off value of 16. Age and the case or control variable were included in all three final models, and interactions between the case or control variable and the other variables present were checked individually in each of the final models. Findings with two-sided *P* values < .05 were considered significant. All data were analyzed using the Statistical Package for Social Science version 15.0 (SPSS Inc., Chicago, IL, United States).

Authorization for the use of information from medical records for research purposes was obtained from the Norwegian Ministry of Health and Social Affairs. The study was approved by the Data Protection Official at Oslo University Hospital, and the Regional Ethics Committee, Region East, Norway (26^th^ March 2004). All participants provided written informed consent. The study was registered at http://www.clinicaltrials.gov, with registration number NCT 00856076.

## Results

The mean gestational age at the index pregnancy was 32.6 weeks (SD 6.0) for the cases, and the mean time from fetal death to assessment was 10.7 years (range 5–18 years, SD 4.0). The majority of the cases had, by invitation from the hospital or on own initiative, received short-term interventions. 76 (75.2%) had a postpartum consultation with the obstetrician, 17 (16.8%) had a consultation with a psychologist/psychiatrist, 54 (53.5%) participated in a bereavement group, 58 (57.4%) had a consultation with the midwife, 25 (24.8%) received follow-up from their general practitioner/regular gynaecologist, 36 (35.6%) had a consultation with a priest/religious counsellor, and 17 (16.8%) had a consultation with other health care personal/hospital staff. Only 9 (8.9%) did not receive any post-partum follow-up.

The time since the fetal death did not differ significantly between the women with a previous IUFD who participated in the study and those who did not. Demographic and clinical factors at the time of the index pregnancy did not differ significantly between participants and nonresponders among cases and controls (data not shown).

The demographic characteristics are presented in Table 1. The cases had a lower level of education and fewer had a household income more than NOK 750 000 per year. They consumed alcohol less frequently, had higher BMI, and more were daily smokers compared with the controls. The cases and controls did not differ on their perceptions of personal finances (very good 24.5% vs 35.9%, good 66% vs 57.6%, poor 9.4% vs 6.5%, *P* = .093), difficulties paying bills (26.4% vs 22.9%, *P* = .474), or numbers who were physically active more than once a week (57.1% vs 59.3%, *P* = .705).

**Table 1:** Demographic and health-related variables for cases and controls

	**Cases n = 106**	**Controls n = 262**	***P*value**
	**n (%)**	**n (%)**	
**Age 2008 (yrs)**			
24–34	11 (10.4)	26 (9.9)	.523
35–39	27 (25.5)	56 (21.4)	
40–44	38 (35.8)	85 (32.4)	
45–57	30 (28.3)	95 (36.3)	
**Living status**			
Married/cohabitating	91 (85.8)	228 (87.0)	.764
Living alone	15 (14.2)	34 (13.0)	
**Education**			
Primary/secondary/high school	25 (23.6)	29 (11.1)	.001
High school + 1–5 years	60 (56.6)	139 (53.1)	
High school + >5 years	21 (19.8)	94 (35.9)	
**Occupational status**			
Working full time (90–100%)	61 (57.5)	168 (64.1)	.239
Not working full time	45 (42.5)	94 (35.9)	
**Household income**			
<750 000 NOK	54 (52.9)	100 (38.5)	.012
≥750 000 NOK	48 (47.1)	160 (61.5)	
**BMI (kg/m**^**2**^**)**			
<25	56 (53.3)	177 (67.8)	.009
≥25	49 (46.7)	84 (32.2)	
**Daily smoking**			
Yes	17 (16.3)	22 (8.4)	.027
No	87 (83.7)	239 (91.6)	
**Alcohol consumption**			
≤1 day per week	82 (78.1)	139 (53.3)	<.001
>1 day per week	23 (21.9)	122 (46.7)	
**Pain**			
Yes	22 (21.8)	33 (12.8)	.035
No	79 (78.2)	224 (87.2)	
**Physically worn out after a day’s work**			
Never/rarely	62 (62.0)	189 (76.5)	.006
Often/always	38 (38.0)	58 (23.5)	
**Mentally worn out after a day’s work**			
Never/rarely	40 (39.6)	142 (55.5)	.007
Often/always	61 (60.4)	114 (44.5)	
**Own health assessment**			
Good	92 (87.6)	252 (96.2)	.002
Poor	13 (12.4)	10 (3.8)	

*NOK*, norwegian kroner (100 NOK = ~13 euros).

The cases had significantly more pregnancies than the controls (4.2 vs 3.0, *P* < .001). The number of live-born children (2.2 vs 2.3, *P* = .866) and the proportion who had experienced miscarriage (38.7% vs 31.0%, *P* = .159) or provoked abortion (22.9 vs 23.0, *P* = .978) did not differ between cases and controls. However, fewer cases felt they had obtained the number of children they wished for (61.0% vs 78.6%, *P* < .001).

The mean number of co-morbid disorders differed significantly between the two groups (1.6 vs 1.2, *P* = .012). The cases reported more frequently pain (21.8% vs 12.8%, *P* = .035), more were physically and mentally exhausted from work, and felt that their health was poor compared with the controls (Table 1). The number of cases and controls reporting sick leave for more than two weeks within the past 12 months did not differ (26.3% vs 18.7%, *P* = .114).

The cases reported slightly lower QOL on the QLI health and functioning subscale (22.3. vs 23.5, *P* = .023). They also scored higher on the CES-D depressed affect subscale (2.0 vs 1.0, *P* = .004) and the CES-D global score (7.0 vs 5.0, *P* = .017) (Table 2). However, the number of cases and controls with a CES-D score above the cut-off value of 16 and the general well-being score did not differ between groups.

**Table 2:** Scores on QLI, GHQ-20, and CES-D for cases and controls

	**Cases**			**Controls**			***P*value**
	**Mean (SD)**	**95% CI**	**Median (IQR)**	**Mean (SD)**	**95% CI**	**Median (IQR)**	
**QLI (0–30)**	n = 104			n = 259			
Health/functioning	22.3 (5.0)	21.3–23.2	22.9 (5.3)	23.5 (4.5)	22.9–24.0	24.1(6.4)	.023
Socioeconomic	22.8 (3.9)	22.1–23.6	23.3 (4.8)	23.4 (4.0)	22.9–23.9	23.9 (5.4)	.224
Psychological/spiritual	22.6 (4.7)	21.6–23.5	23.0 (6.2)	22.7 (4.4)	22.1–23.2	23.1(5.6)	.798
Family	26.2 (4.4)	25.4–27.1	27.6 (6.0)	26.6 (4.2)	26.1–27.1	27.3(4.5)	.383
Global QLI	22.9 (4.0)	22.2–23.7	23.5 (4.6)	23.7 (3.9)	23.2–24.1	24.2 (5.4)	.108
**GHQ-20 (0–60)**	n = 103			n = 259			
Global GHQ-20	20.6 (6.8)	19.3–22.0	20.0 (9.0)	19.4 (6.5)	18.6–20.1	18.0 (7.0)	.094
	**Median (IQR)**			**Median (IQR)**			
**CES-D**	n = 103			n = 259			
Depressed affect (0–21)	2.0 (5.0)			1.0 (3.0)			.004
Positive affect (0–12)	2.0 (5.0)			2.0 (4.0)			.220
Somatic affect (0–21)	3.0 (4.0)			2.0 (5.0)			.094
Interpersonal (0–6)	0.0 (0.0)			0.0 (0.0)			.322
Global CES-D (0–60)	7.0 (10.5)			5.0 (9.0)			.017
	**N**	**%**		**N**	**%**		
Value ≥16	18	17.1		41	15.8		.747

*SD*, standard deviation; *CI*, confidence interval; *IQR*, interquartile range.

As shown in Tables 3 and 4, QOL and depression did not differ between cases and controls after adjusting for possible confounders in the multivariable analyses. The global QLI score was associated with income, subjective judgment of one’s health, mental exhaustion from work, and co-morbidity. Miscarriage was significantly associated with global QLI (*P* = .022). The global CES-D score was associated with income, subjective judgment of one’s health, and physical exhaustion from work. GHQ-20 was not associated with previous IUFD in the multivariable analyses (*P* = 0.70) but was significantly related to subjective judgment of one’s health (*P* < 0.001), mental exhaustion from work (*P* = 0.002), and co-morbidity (*P* = 0.001). There were no significant interactions in any of these models.

**Table 3:** Multivariable analysis of quality of life (global QLI)

	**Univariable**			**Multivariable**		
	**β**	**95% CI**	***P*value**	**β**	**95% CI**	***P*value**
Case (ref: control)	−0.73	−1.62–0.16	.108	0.17	−0.64–0.98	0.674
Age in 2008 (yr)	−0.00	−0.08–0.07	.925	−0.05	−0.12–0.01	.124
Primary, secondary, high school (ref: 1–5 year postsecondary education)	−0.94	−2.13–0.25	.122			
>5 year postsecondary school (ref: 1–5 year postsecondary education)	0.82	−0.08–1.71	.075			
Household income ≥750 000 NOK (ref: <750 000 NOK)	2.22	1.43–3.01	<.001	1.74	1.00–2.48	<.001
Overweight ≥25 (ref: BMI <25)	−1.10	−1.94–−0.27	.010			
Daily smoker (ref: not daily smoker)	−1.42	−2.74–−0.10	.035			
Alcohol consumption, more than once per week (ref: once a week or less)	0.42	−0.41–1.25	.323			
Co-morbidity	−0.92	−1.25–−0.60	<.001	−0.66	−0.97–−0.35	<.001
Pain (ref: no pain)	−2.90	−3.99–−1.81	<.001			
Sick leave two weeks or more (ref: <2 weeks)	−2.10	−3.07–−1.14	<.001			
Often or always physically exhausted from work (ref: never/rarely)	−2.74	−3.62–−1.86	<.001	−0.79	−1.70–0.12	.087
Often or always mentally exhausted from work (ref: never/rarely)	−2.60	−3.37–−1.83	<.001	−1.45	−2.24–−0.65	<.001
Poor health (ref: good)	−5.61	−7.17–−4.05	<.001	−3.69	−5.33–−2.04	<.001
Miscarriage (ref: no miscarriage)	0.58	−0.275–1.434	.183	0.89	0.13–1.65	.022

*CI*, confidence interval; *NOK*, norwegian kroner (100 NOK = ~13 euros).

Interaction is not present in the model.

**Table 4:** Multivariable analysis of depression (global CES-D dichotomised at a cut-off value of 16)

	**Univariable**			**Multivariable**		
	**OR**	**95% CI**	P **value**	**aOR**	**95% CI**	***P*value**
Case (ref: control)	1.11	0.60–2.03	.747	0.77	0.37–1.57	.465
Age in 2008 (yr)	1.00	0.95–1.05	.944	1.01	0.96–1.07	.692
Primary, secondary, high school (ref: 1–5 year postsecondary education)	1.51	0.72–3.18	.280			
>5 year postsecondary school (ref: 1–5 year postsecondary education)	0.77	0.40–1.50	.447			
Household income ≥750 000 NOK (ref: <750 000 NOK)	0.34	0.19–0.60	<.001	0.41	0.21–0.77	.006
Overweight ≥25 (ref: BMI <25)	1.14	0.64–2.03	.648			
Daily smoker (ref: not daily smoker)	2.40	1.12–5.17	.025			
Alcohol consumption, more than once per week (ref: once a week or less)	0.83	0.46–1.48	.526			
Co-morbidity	1.40	1.13–1.73	.002			
Pain (ref: no pain)	3.01	1.55–5.84	.001			
Sick leave two weeks or more (ref: <2 weeks)	2.88	1.53–5.42	.001			
Often or always physically exhausted from work (ref: never/rarely)	3.71	2.03–6.78	<.001	2.34	1.18–4.63	.015
Often or always mentally exhausted from work (ref: never/rarely)	2.35	1.28–4.31	.006			
Poor health (ref: good)	10.23	4.18–25.02	<.001	6.13	2.11–17.77	.001
Miscarriage (ref: no- miscarriage)	0.70	0.38–1.31	.264			

*CI*, confidence interval; *OR*, odds ratio; *aOR*, adjusted odds ratio; *NOK*, norwegian kroner (100 NOK = ~13 euros).

Interaction is not present in the model.

There were no significant differences in global QLI, GHQ-20 or global depression when comparing women who experienced fetal death less than 10 years ago and more than 10 years ago (data not shown).

## Discussion

The women in our study scored somewhat better on QLI than women in the general Swedish population [17], showing that their quality of life is good. The global QLI and QLI subscale values among the controls in our study are quite similar to those obtained in two other studies conducted in Norway each using a control group of women [18,19]. Women with a history of IUFD 5–18 years previously scored slightly lower on health-related QOL and slightly higher on the CES-D subscale depressed affect and global CES-D. These relatively small differences are unlikely to be of clinical significance. The *P* values for these outcomes were borderline and there were no significant differences in global QOL, well-being and global depression when adjusting for socioeconomic and health related factors. Our findings suggest that IUFD has no major impact on women’s long-term QOL, well-being, or risk of experiencing depression symptoms. Previous studies have found negative associations [1,2,5,6], but these studies were limited by much shorter observation periods after the event. It is possible that the much longer time since IUFD in our study reduced negative thoughts, emotions, and the risk of lower QOL. The short-term interventions that were conducted among the majority of the cases may have contributed to this positive outcome. Surprisingly, miscarriage predicted a higher score in the multivariable analysis of global QLI. However, the *P* value would not remain significant when correcting for the high number of analyses performed.

To our knowledge, the relationship between a previous IUFD and long-term QOL has not been investigated previously, although earlier studies have found that the level of psychological distress triggered by an IUFD decreases over time [6,7,20]. The assumption that possible psychological effects of IUFD have normalized within 5–18 years is consistent with what was reported by Cacciatore et al., that the level of depression among women with fetal death decreases after one year [6], and Turton et al. [7] that there were no difference in the levels of PTSD and depression between cases and controls 6–8 years after the subsequent birth. We found lower socioeconomic status and educational level among the cases, findings that are also consistent with those of previous studies [21-23]. Lower socioeconomic status has consistently been associated with an increased risk of IUFD, but the reasons for this association remain unknown [24,25].

There are inherent limitations in our study. Because of the many statistical analyses conducted on these data, there is a risk of type I statistical error and the estimates of *P* values > .001 should be interpreted with caution. The response rates in the present study were 31% and 80%, respectively, for women having experienced an IUFD and women having live-born children only. The controls were invited from an already selected population, resulting in an uneven response rate between cases and controls. Potential responders are more likely to participate if the study is concerned with an issue particularly salient to their lives [26]. Presumably, patients and controls from the general population have a lower threshold for declining studies than do patients during active treatment. An IUFD is a potentially traumatizing experience that women may not wish to be reminded of many years later. A low response rate increases the risk of selection bias, and it is possible that women with negative long-term outcomes more frequently declined participation in the study. On the other hand, it is also possible that women who had adapted to the incident had little interest in participating. We found no significant differences between responders and non-responders in available clinical and demographic data. Even though the response rate was low, the bias testing supports the main finding that there was no major influence of IUFD on long-term QOL and psychological distress at a group level. A strength of this study is that the data were gathered from two university hospitals that cover a substantial proportion of women giving birth in this part of Norway. We used acknowledged and well-validated instruments to assess the level of QOL, well-being, and depression, and our data comprise co-morbidity, sociodemographic, and pregnancy-related determinants likely to affect QOL, well-being, and depression. Because patients with lower sociodemographic status and educational level are less likely to participate in scientific studies [26], they were probably overrepresented among the non-responders, but this probably accounts for both cases and controls. However, the main conclusion that there was no long-term impact of IUFD would probably have remained the same. We therefore argue that the findings can be generalized to other women who have experienced an IUFD many years previously.

## Conclusions

Long-term QOL, well-being, and risk of depressive symptoms at group level were not affected by IUFD in our study, in which the majority of the women had received short-term interventions. This is important knowledge for health care personnel who provide care and guidance to women in this situation. However, as previous studies have shown that many women suffer shortly after the event it is important to continue with general good care in the acute phase. Future research in this patient group would benefit from cohort studies with long-term follow-up to establish certainties about the consequences of IUFD and to identify possible subgroups in need of special care.

## Abbreviations

BMI: Body mass index; CES-D: Center of epidemiological studies depression scale; IUFD: Intrauterine fetal death; GHQ-20: General health questionnaire 20; NOK: Norwegian kroner; QLI: Quality of life index; QOL: Quality of life; PTSD: Post traumatic stress disorder; SD: Standard deviation; VIP: The venous thromboembolism in pregnancy study.

## Competing interests

The authors declare that they have no competing interests.

## Authors’ contributions

IKG performed the analyses, interpreted the results and wrote the main draft of the manuscript. LBH designed the original study, collected the data, helped to interpret the results and critically revised the manuscript for important intellectual content. EMJ designed the original study, helped to interpret the results and critically revised the manuscript for important intellectual content. PMS designed the original study, helped interpret the results, revised the manuscript for important intellectual content and supervised the study. ØE helped design the study, helped with the statistical analyses, helped to interpret the results and critically revised the manuscript for important intellectual content. All authors read and approved the final version of the manuscript.

## Pre-publication history

The pre-publication history for this paper can be accessed here:

http://www.biomedcentral.com/1471-2393/12/43/prepub
